# Survival prediction of kidney renal papillary cell carcinoma by comprehensive LncRNA characterization

**DOI:** 10.18632/oncotarget.22732

**Published:** 2017-11-28

**Authors:** Huihua Lan, Jianghui Zeng, Gang Chen, Huayi Huang

**Affiliations:** ^1^ Department of Laboratory Medicine, The People's Hospital of Guangxi Zhuang Autonomous Region, Nanning, Guangxi, China; ^2^ Department of Laboratory Medicine, The Third Affiliated Hospital of Guangxi Medical University/The Second People’s Hospital of the City of Nanning, Nanning, Guangxi, China; ^3^ Department of Pathology, The First Affiliated Hospital of Guangxi Medical University, Nanning, Guangxi, China; ^4^ Department of Surgical Oncology, Roswell Park Cancer Institute, Buffalo, New York, USA

**Keywords:** LncRNA, the cancer genome atlas (TCGA), renal papillary cell carcinoma, prognosis

## Abstract

Kidney renal papillary cell carcinoma (KIRP) accounts for 10%–15% of renal cell carcinoma (RCC), patients with KIRP tend to have a poor prognosis, and there was a lack of effective prognostic indicators for this type of cancer. Currently, owing to the availability of The Cancer Genome Atlas (TCGA), long non-coding RNAs (LncRNAs) have been discovered to indicate a prognostic value in some tumors. In that regard, we analyzed lncRNA-sequencing data of KIRP in TCGA, and among 780 differentially-expressed lncRNAs, we selected 37 lncRNAs which were able to assist the prognosis. In addition, by using the multivariate cox regression analysis, the prognosis index (PI) that consisted of 7 lncRNAs (including AFAP1-AS1, GAS6-AS1, RP11-1C8.7, RP11-21L19.1, RP11-503C24.1, RP11-536I6.2, and RP11-63A11.1) could predict the progression and outcomes of KIRP with accuracy. More importantly, the PI was considered an independent indicator for prognostication of KIRP. Moreover, having categorized patients with KIRP into cohorts of high risk and low risk, according to the PI, we found that the key genes and pathways varied in these two groups. Overall, these LncRNAs, especially the PI, may be conceived as biomarkers and helpful for determining the different pathological stages for KIRP patients. However, their biological functions need to be further confirmed.

## INTRODUCTION

Kidney cancer is a neoplasm with heterogeneity, of which epithelial renal cell carcinoma (RCC) represents the major proportion. Morphologically, RCC includes various histological subtypes—kidney renal clear cell carcinoma (KIRC), kidney renal papillary cell carcinoma (KIRP), and malignancies of chromophobe, collecting duct, and subtypes with classification. Of these subtypes, KIRP ranks second in terms of morbidity rate, comprising 10%–15% of cases, following KIRC with incidence of 75%–80% [1–5].

Approximately 30% of RCC patients have been found to exhibit distant metastasis when diagnosed, thus the prognosis of RCC remains poor. Clinically, patients with other RCC subtypes tend to have desirable outcomes; however, patients with KIRP were more likely to experience obviously worse clinical courses [1, 6, 7].

Currently, several biomarkers for KIRC that have been detected include von Hippel-Lindau (VHL) [8–11], vascular endothelial growth factor (VEGF) [12–15], carbonic anhydrase IX (CAIX) [16–18], and hypoxia-inducible factor 1 alpha/2 alpha (HIF1a/2a) mutations [19–21], some of which were able to forecast the medical effectiveness and outcomes. Despite that, there have been scarce studies into the molecular biomarkers of KIRP for the prediction of therapeutic efficacy and prognostication. Long non-coding RNAs (LncRNAs) were considered to play a vital role in tumorigenesis and progression, exhibited by their capability to predict the patients’ outcomes [22–26]. Nonetheless, studies found that only a small number of lncRNAs could foretell the development and prognosis of KIRP. Hence, it is important to seek novel molecular biomarkers of lncRNAs with prognostic value for KIRP, which would facilitate the understanding of the pathogenesis of KIRP and be helpful in prognosis evaluation.

The Cancer Genome Atlas (TCGA) has rendered the results of KIRP available for researchers [27–30], which updated the knowledge towards the understanding of lncRNA-based diseases. Nevertheless, there is no report regarding the clinical significance of lncRNAs and the differences among lncRNAs based on the TCGA data so far. Consequently, mRNA-Seq data, including lncRNAs, of patient samples with KIRP collected from TCGA data were set to recognize some key lncRNAs that were related to clinical manifestations. We particularly focused on the ability of prognostic evaluation of these lncRNAs, and investigated the clinical significance by selecting seven lncRNAs with high prognostic potential to form a pool. These key lncRNAs would show clinical significance and play a role in the initiation and development of KIRP.

## RESULTS

### Differentially-expressed lncRNAs (DELs)

**Figure 1:**
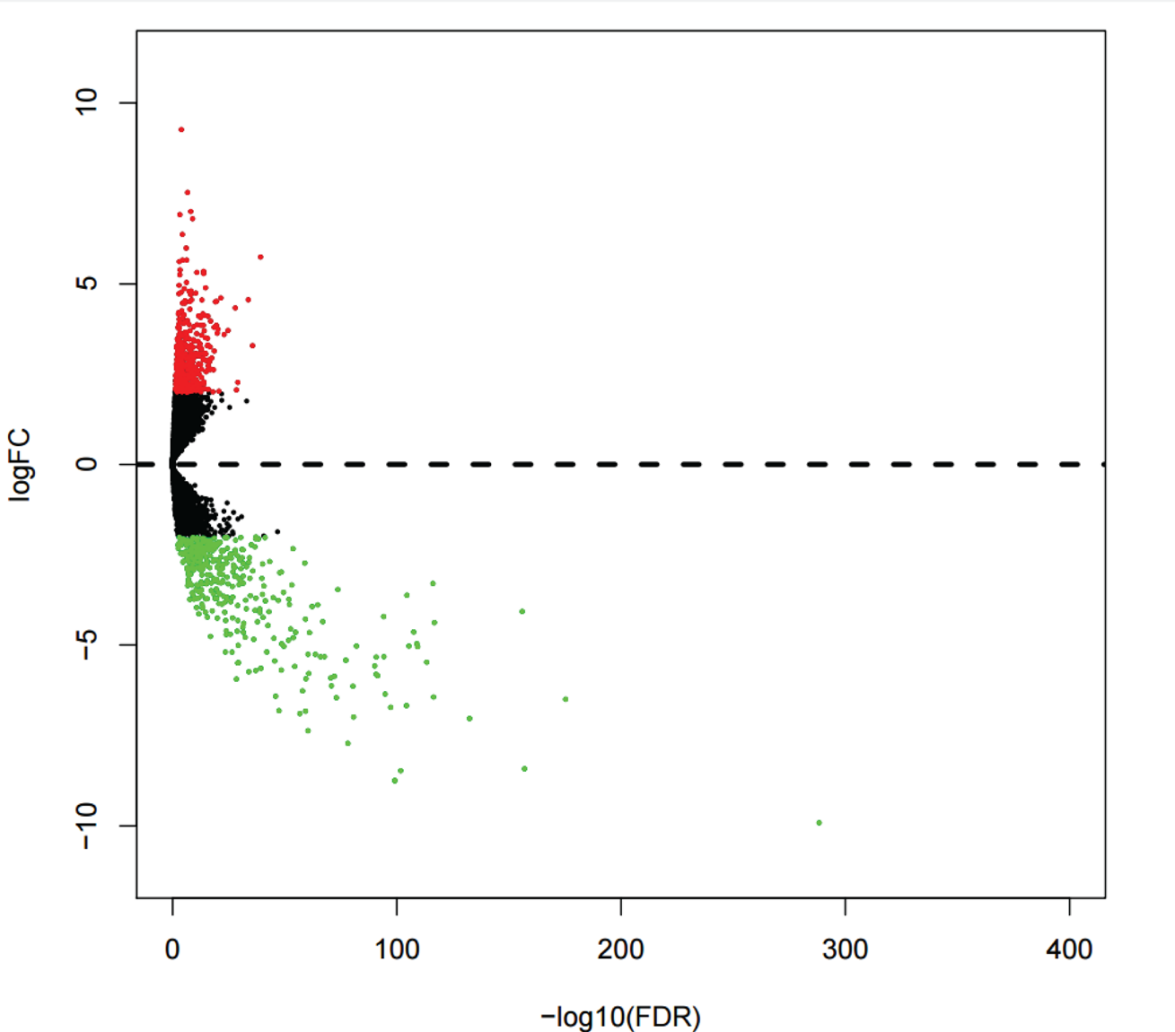
Volcano plot of differentially expressed lncRNAs (DELs) in kidney renal papillary cell carcinoma (KIRP) DELs filtered using the edgeR package with Padj < 0.05 and |log_2_FC| > 2. Volcano plot was drawn by the gplots package.

**Figure 2:**
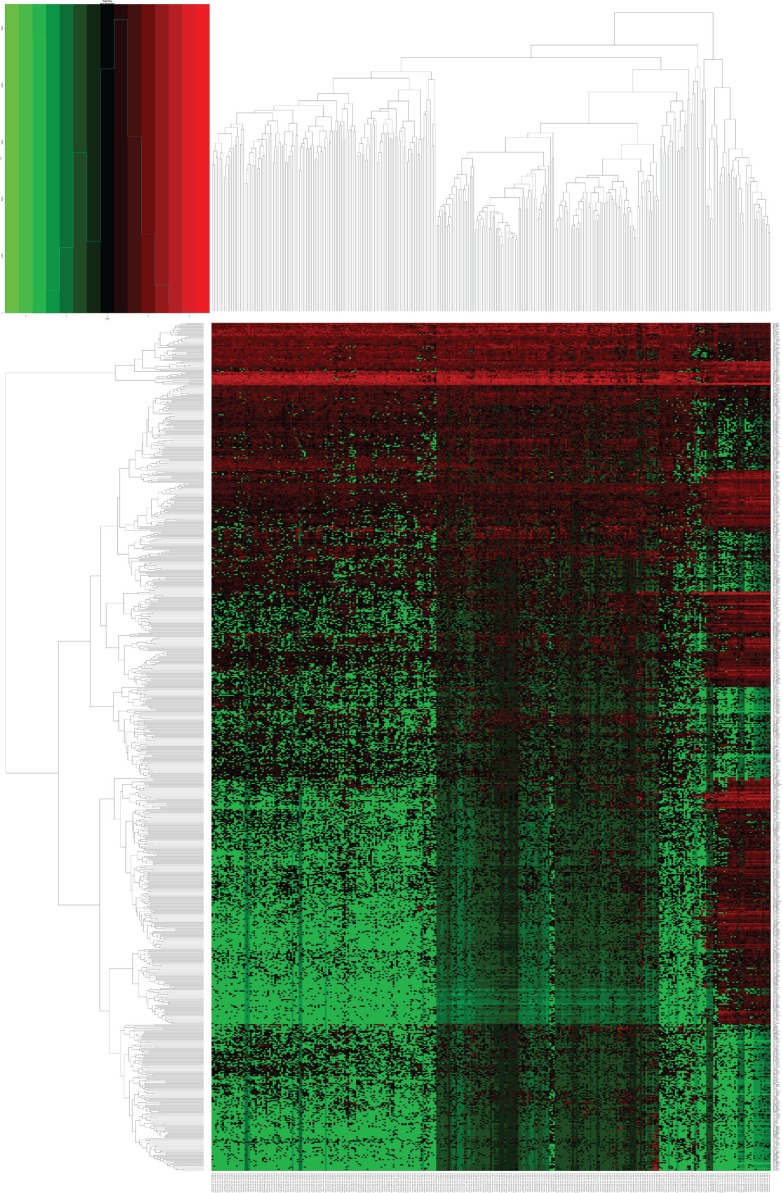
Heatmap of differentially expressed lncRNAs (DELs) in kidney renal papillary cell carcinoma (KIRP) DELs identified using the edgeR package with Padj < 0.05 and |log_2_FC| > 2. Heatmap was generated by the gplots package.

Analysis of the DELs was conducted to compare the expressions of 13,198 lncRNAs between KIRP and normal kidney tissues. A total of 780 DELs (Figures 1, 2) were collected by EdegR. Then we eliminated cases without adequate data on survival duration, and finally, we obtained 271 DELs for further exploration.

### Construction of DEL-based prognostic signature

**Figure 3:**
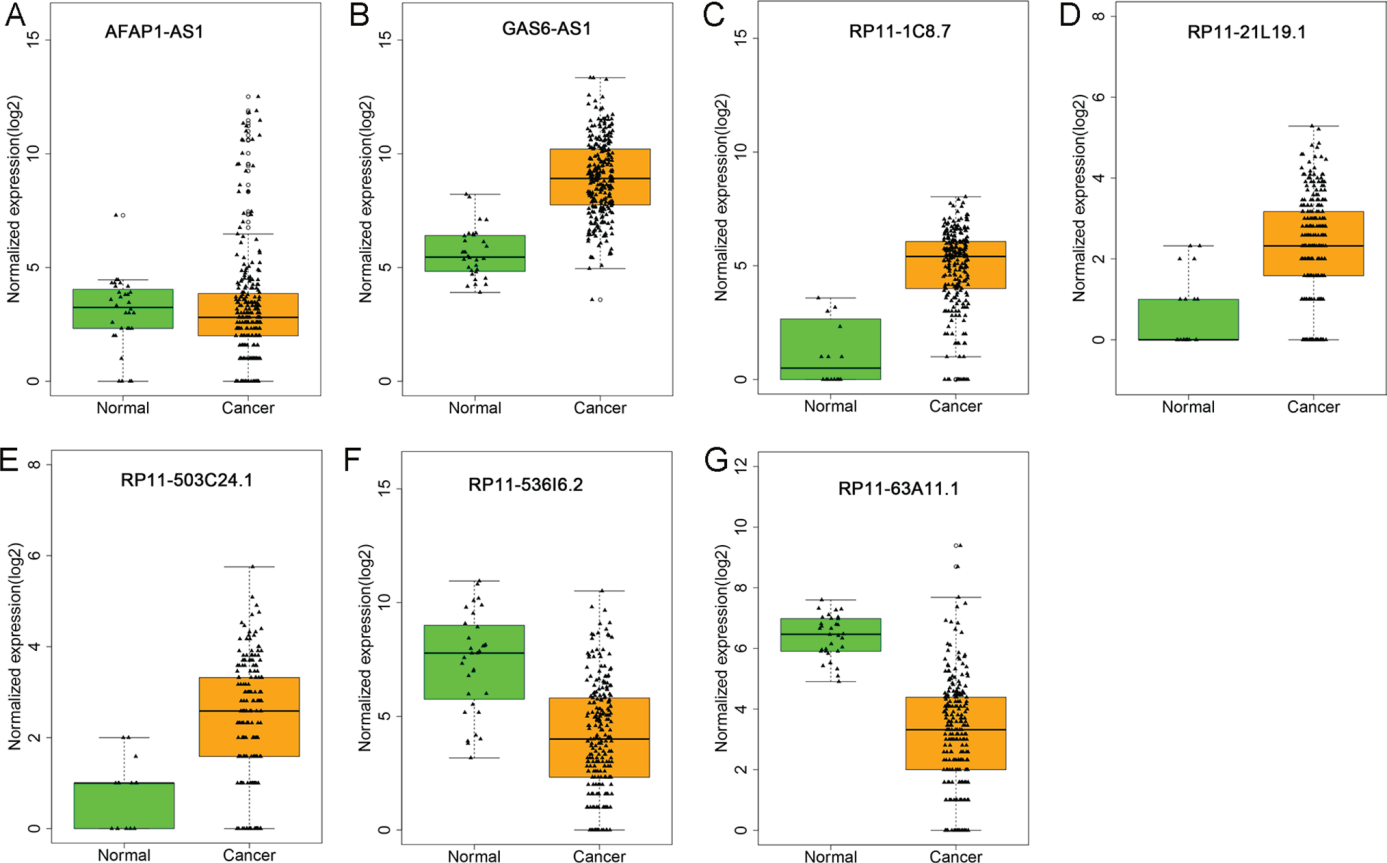
Different expression of the seven lncRNAs between kidney renal papillary cell carcinoma (KIRP) and normal tissues The boxplots were generated by R language. Statistical differences were analyzed using the two-sample *t*-test. Green column indicated normal tissue and dark yellow column showed KIRP tissues. (**A**) AFAP1-AS1; (**B**) GAS6-AS1; (**C**) RP11-1C8.7; (**D**) RP11-21L19.1; (**E**) RP11-503C24.1; (**F**) RP11-536I6.2; (**G**) RP11-63A11.1.

**Figure 4:**
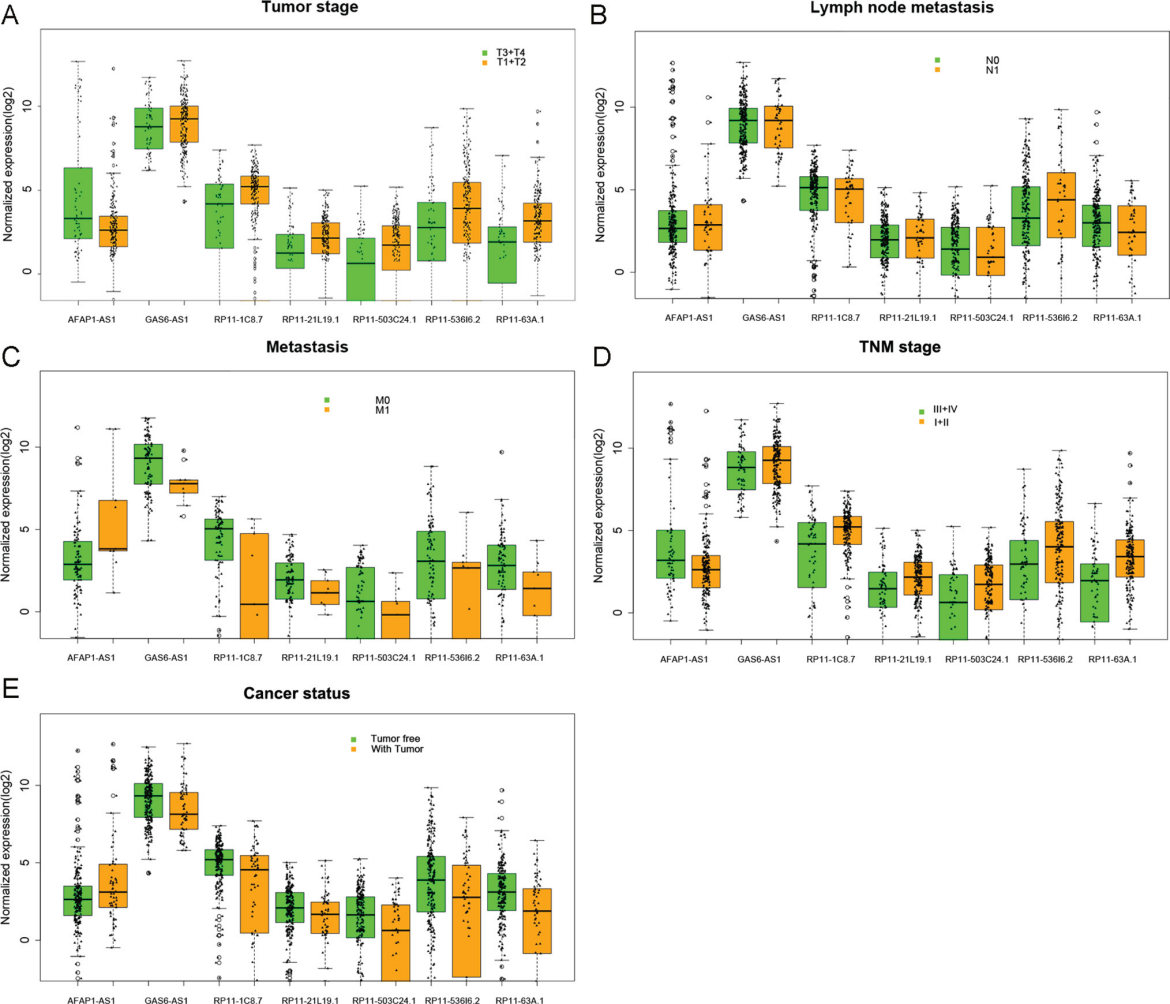
The relationship between the expression of major lncRNAs and clinical pathological features in kidney renal papillary cell carcinoma (KIRP) The boxplots were drawn by R language. Statistical differences were analyzed by the two-sample *t*-test. These major lncRNAs statistical significance differences were noted in several clinical pathological features: (**A**) tumor stage (T3/T4 vs. T1/T2), (**B**) lymph node metastasis (N0 *vs*. N1), (**C**) distant metastasis (M0 vs. M1), (**D**) TNM stage (III/IV *vs*. I/II), and (**E**) cancer status (tumor free *vs*. tumor). The X axis shows different lncRNAs, and the Y axis indicates the normalized expression (log2).

**Figure 5:**
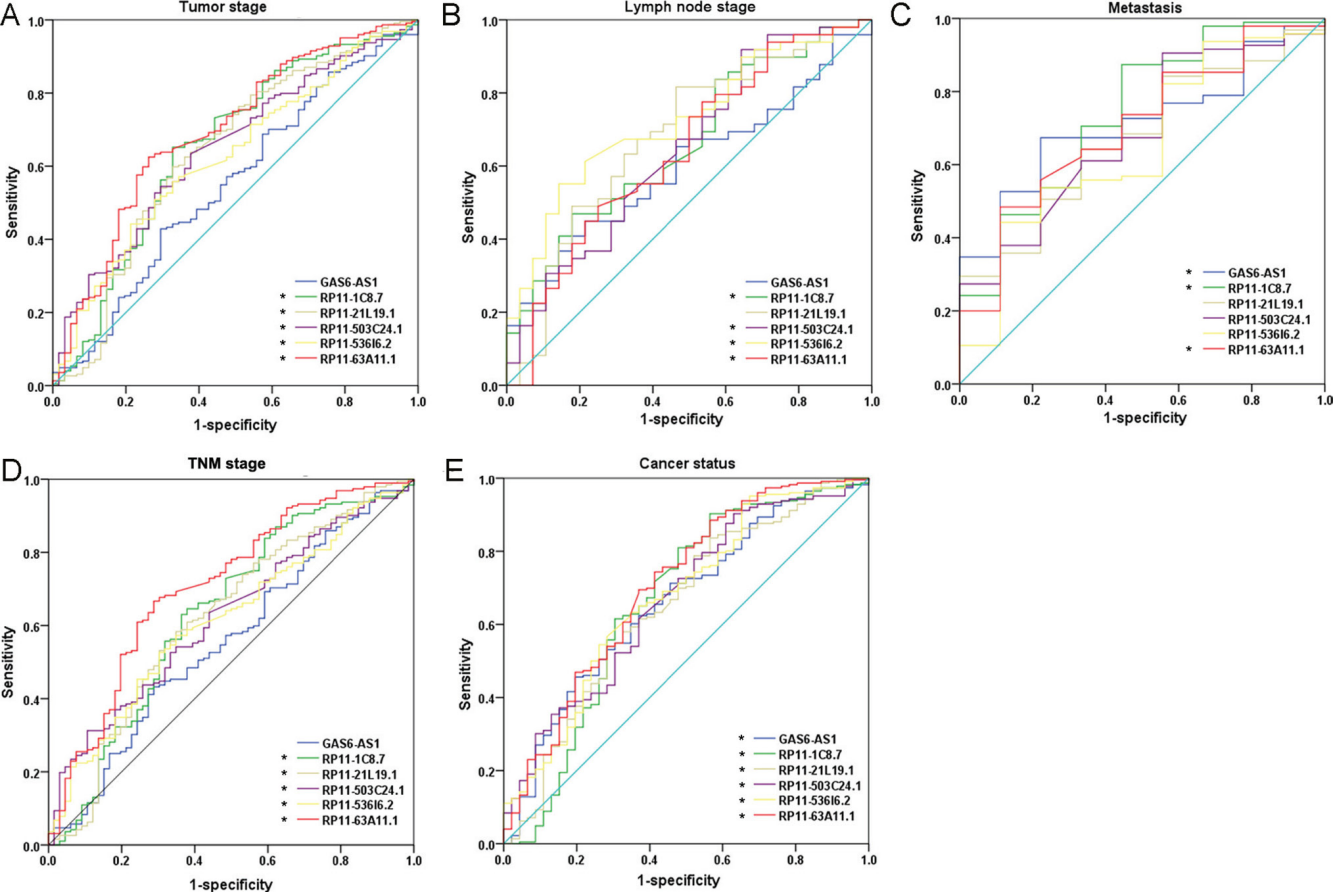
The predictive effect of lncRNAs on clinical progression of kidney renal papillary cell carcinoma (KIRP) by receiver operating characteristic (ROC) The ROC curves were generated by SPSS and used to estimate the predicted value of each lncRNA for the cancer progress including: (**A**) advanced tumor stages (T3–4), (**B**) lymph node metastasis positive, (**C**) metastasis, (**D**) higher TNM stages (III–IV), and (**E**) cancer status (with tumor). ^*^P < 0.05 for AUC of each lncRNA.

It was demonstrated by the univariate Cox proportional hazards regression that 37 of these 271 differentially-expressed lncRNAs displayed remarkable prognostic value. Then multivariate Cox proportional hazards regression analysis confirmed that seven differentially-expressed lncRNAs showed prognostic significance for KIRP, containing actin filament associated protein 1 antisense RNA1(AFAP1-AS1), GAS6 antisense RNA 1 (GAS6-AS1), RP11-1C8.7, RP11-21L19.1, RP11-503C24.1, RP11-536I6.2, and RP11-63A11.1 (Table 1 and Figure 3). The relationship between the expression of major lncRNAs and clinicopathological features in KIRP are shown in Figure 4. The expression of the seven lncRNAs could also forecast the development of KIRP (Table 2 and Figure 5). The prognosis index (PI) formula for overall survival prediction was as follows:

**Table 1:** Detailed information of seven prognostic lncRNAs in KIRP

lncRNA	Esenble ID	log_2_FC	FER	β (Cox)	SE	*P*-value	Exp (B)	Lower	Upper
AFAP1-AS1	ENSG00000272620	3.213267784	3.85E-05	0.195	0.071	0.006	1.215	1.056	1.398
GAS6-AS1	ENSG00000233695	3.709316256	1.91E-25	–0.474	0.126	<0.001	0.622	0.487	0.796
RP11-1C8.7	ENSG00000271830	4.611213539	3.09E-22	–0.243	0.071	0.001	0.784	0.682	0.901
RP11-21L19.1	ENSG00000254418	2.34604761	3.44E-11	–0.204	0.083	0.013	0.815	0.693	0.959
RP11-503C24.1	ENSG00000234768	2.277851897	1.30E-06	0.335	0.112	0.003	1.397	1.123	1.740
RP11-536I6.2	ENSG00000255021	–3.06775197	1.59E-17	–0.251	0.074	0.001	0.778	0.674	0.899
RP11-63A11.1	ENSG00000250781	–2.201710907	3.19E-12	–0.244	0.058	<0.001	0.783	0.699	0.878

**Table 2:** The association between these seven lncRNAs and clinical features of KIRP patients

Factor		AFAP1–AS1	GAS6–AS1	RP11–1C8.7	RP11–21L19.1	RP11–503C24.1	RP11–536I6.2	RP11–63A11.1
**Tumor stage (T3–4/T1–2)**	***t*-test**	3.737	–1.052	–3.225	–2.733	–3.244	–3.042	–4.910
	***P***	**<0.001**	**0.294**	**0.002**	**0.008**	**0.001**	**0.003**	**<0.001**
	**AUC**	0.369	0.550	0.654	0.641	0.647	0.626	0.695
	***P***	**0.002**	**0.227**	**<0.001**	**0.001**	**<0.001**	**0.003**	**<0.001**
**Lymph node metastasis (N1/N0)**	***t*-test**	–2.965	1.540	2.146	2.290	2.017	3.392	2.117
	***P***	**0.005**	**0.128**	**0.037**	**0.025**	**0.049**	**0.001**	**0.038**
	**AUC**	0.332	0.601	0.662	0.681	0.647	0.724	0.642
	***P***	**0.014**	**0.144**	**0.018**	**0.009**	**0.032**	**0.001**	**0.039**
**Metastasis (M1/M0)**	***t*-test**	–2.064	2.083	2.235	1.183	1.579	1.652	1.789
	***P***	**0.070**	**0.040**	**0.053**	**0.240**	**0.118**	**0.102**	**0.077**
	**AUC**	0.272	0.713	0.740	0.671	0.684	0.653	0.702
	***P***	**0.024**	**0.035**	**0.018**	**0.090**	**0.069**	**0.131**	**0.046**
**TNM stage (III–IV/I–II))**	***t*-test**	3.365	–1.251	–2.936	–2.601	–2.587	–2.653	–5.482
	***P***	**0.001**	**0.212**	**0.004**	**0.011**	**0.010**	**0.008**	**<0.001**
	**AUC**	0.380	0.557	0.634	0.624	0.624	0.614	0.710
	***P***	**0.004**	**0.171**	**0.001**	**0.003**	**0.003**	**0.006**	**<0.001**
**Cancer status (with tumor/tumor free)**	***t*-test**	–3.058	3.308	3.451	2.790	3.520	3.982	4.384
	***P***	**0.003**	**0.001**	**0.001**	**0.007**	**0.001**	**<0.001**	**<0.001**
	**AUC**	0.361	0.661	0.661	0.649	0.670	0.675	0.705
	***P***	**0.048**	**0.001**	**0.001**	**0.001**	**<0.001**	**<0.001**	**<0.001**

**Figure 6:**
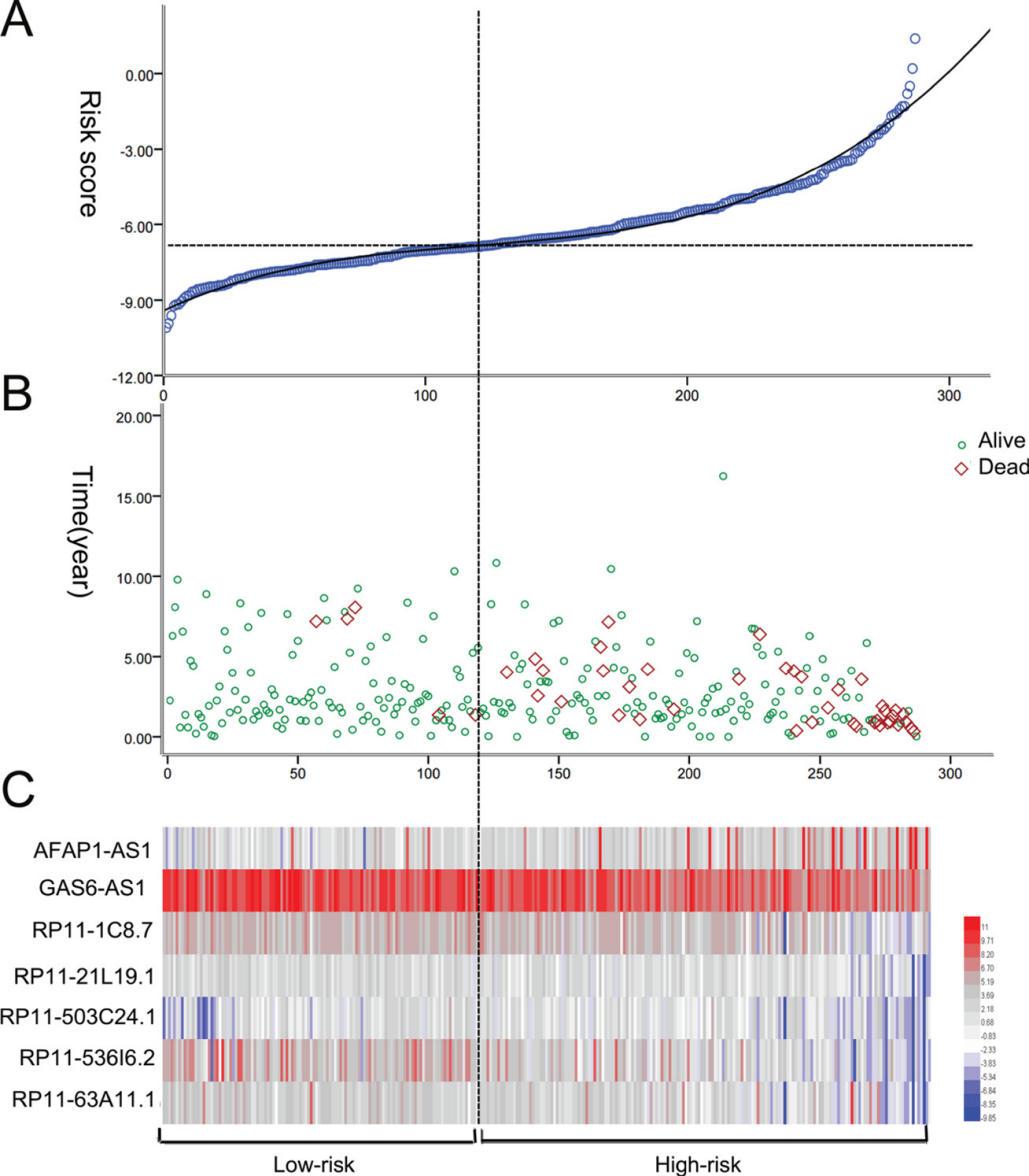
The risk score analysis of kidney renal papillary cell carcinoma (KIRP) patients (**A**) LncRNA predictive risk score distribution; (**B**) The survival status of KIRP cases; (**C**) Heatmap of the seven lncRNAs expression profiles in KIRP. From blue to red indicates a trend from low expression to high expression.

**Figure 7:**
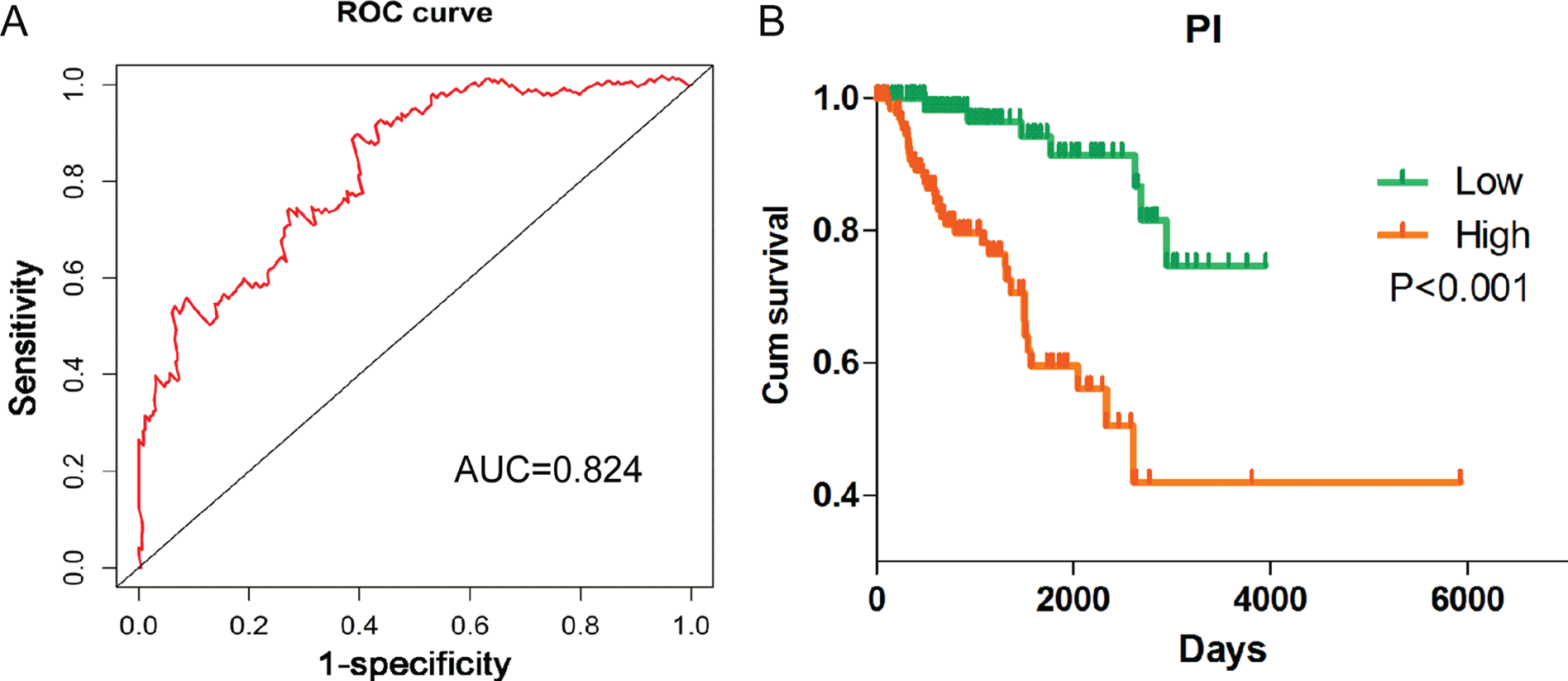
Survival ROC curve and Kaplan–Meier curve for the seven lncRNAs signature in kidney renal papillary cell carcinoma (KIRP) (**A**) Survival ROC curve was generated by Survival ROC package of R language. Time-dependent ROC curves analysis relies on the 7-lncRNAs signatures for survival prediction. (**B**) Kaplan–Meier curve was drawn by GraphPad Prism. Kaplan-Meier survival curve showed overall survival outcomes by relative high-risk and low-risk patients.

PI = 0.195^*^exp_AFAP1–AS1_–0.474^*^exp_GAS6-AS1_–0.243^*^_expRP11-1C8.7_–0.204^*^exp_RP11–21L19.1_ + 0.335^*^exp_RP11–503C24.1_–0.251^*^_expRP11–536I6.2_–0.244^*^exp_RP11–63A11.1_. The patients with KIRP were classified into high risk and low risk groups according to the PI (Figure 6). Also the PI, to a large extent, was able to foretell the 5-year survival of KIRP patients, with the area under the receiver operating characteristic curve (AUC) value being 0.824 (Figure 7A). Moreover, Kaplan-Meier (K-M) curves showed that the average survival time of patients in the high risk group was 109.4 months, which was noticeably shorter than that of their low risk counterparts (117.3 months, *P* < 0.001, Figure 7B).

**Figure 8:**
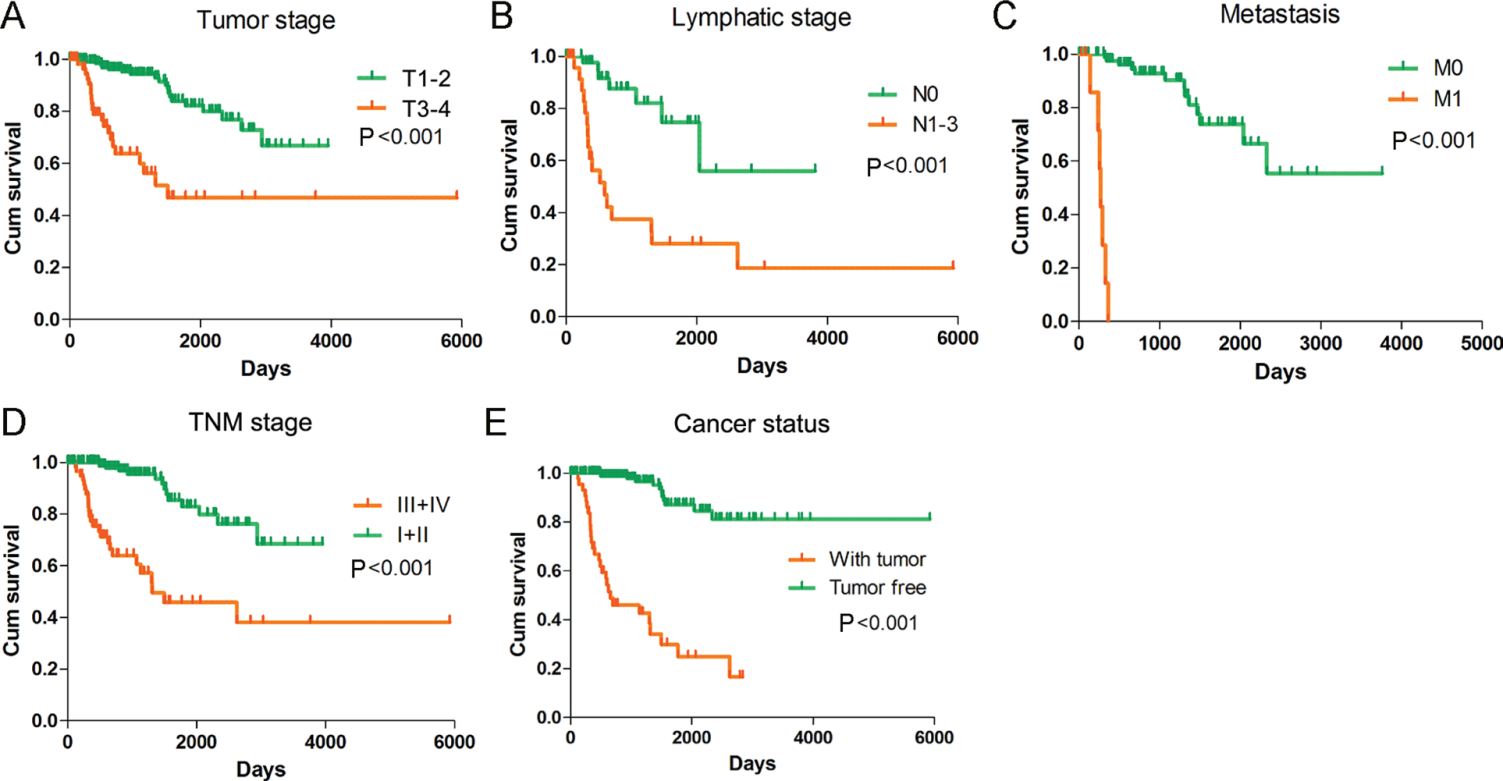
Kaplan-Meier survival curves in subgroup analyses of different clinical factors Kaplan–Meier curves were generated by GraphPad Prism. (**A**) Tumor stage (HR = 5.074, *P* < 0.001); (**B**) Lymphatic stage (HR = 4.994, *P* < 0.001); (**C**) Metastasis (HR = 114.966, *P* < 0.001); (**D**) TNM stage (HR = 6.473, *P* < 0.001); (**E**) Cancer status (HR = 15.389, *P* < 0.001).

Meanwhile, we also measured the prognosis value of various clinical parameters with PI. By the univariate Cox proportional hazards regression, we discovered that a variety of parameters were closely related to undesirable prognosis of KIRP patients (Table 3). Yet, the multivariate Cox proportional hazards regression unveiled that only distant metastasis could be an independent prognosis indicator for KIRP (Table 3). The K-M curves of the clinical features were depicted in Figure 8.

**Table 3:** Univariate and multivariate cox analyses for the prognostic value of clinical features of KIRP patients

Variables	Univariate				Multivariate			
	*P*	HR	LL	UL	*P*	HR	LL	UL
PI (high-risk/low-risk)	<0.001	2.163	1.813	2.580	0.002	2.400	1.374	4.192
Gender (female/male)	0.149	0.617	0.320	1.190	0.548	1.743	0.284	10.706
Age (>60/<60)	0.881	0.955	0.527	1.733	0.751	1.284	0.274	6.023
T (T3–4/T1–2)	<0.001	5.074	2.765	9.310	0.829	0.799	0.104	6.131
N (N1/N0)	<0.001	4.994	2.058	12.118	0.254	0.300	0.038	2.376
M (M1/M0)	<0.001	114.966	22.481	587.924	0.008	42.852	2.628	698.744
Cancer status (with tumor/tumor free)	<0.001	15.389	7.812	30.316	0.842	1.290	0.105	15.842
TNM stage (III–IV/I–II)	<0.001	6.473	3.362	12.462	0.903	1.205	0061	23.920

**Figure 9:**
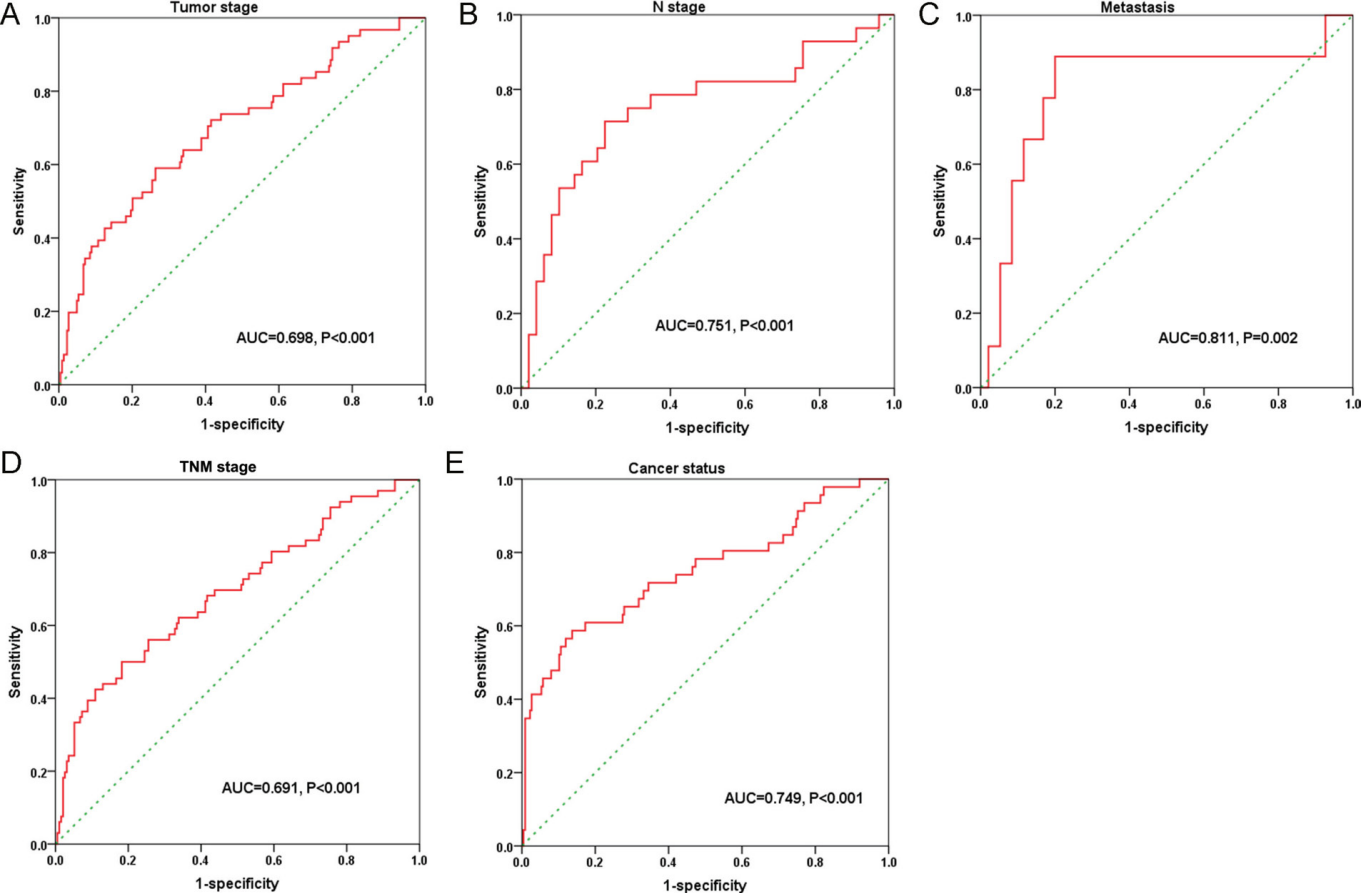
Predictive value of the risk score for clinical features according to the receiver operating characteristic (ROC) curves The ROC curves were drawn by SPSS. (**A**) Tumor stage (AUC = 0.698, *P* < 0.001); (**B**) *N* stage (AUC = 0.751, *P* < 0.001); (**C**) Distant metastasis (AUC = 0.811, *P* < 0.001); (**D**) TNM stage (AUC = 0.691, *P* < 0.001); (**E**) Cancer status (AUC = 0.749, *P* < 0.001).

**Figure 10:**
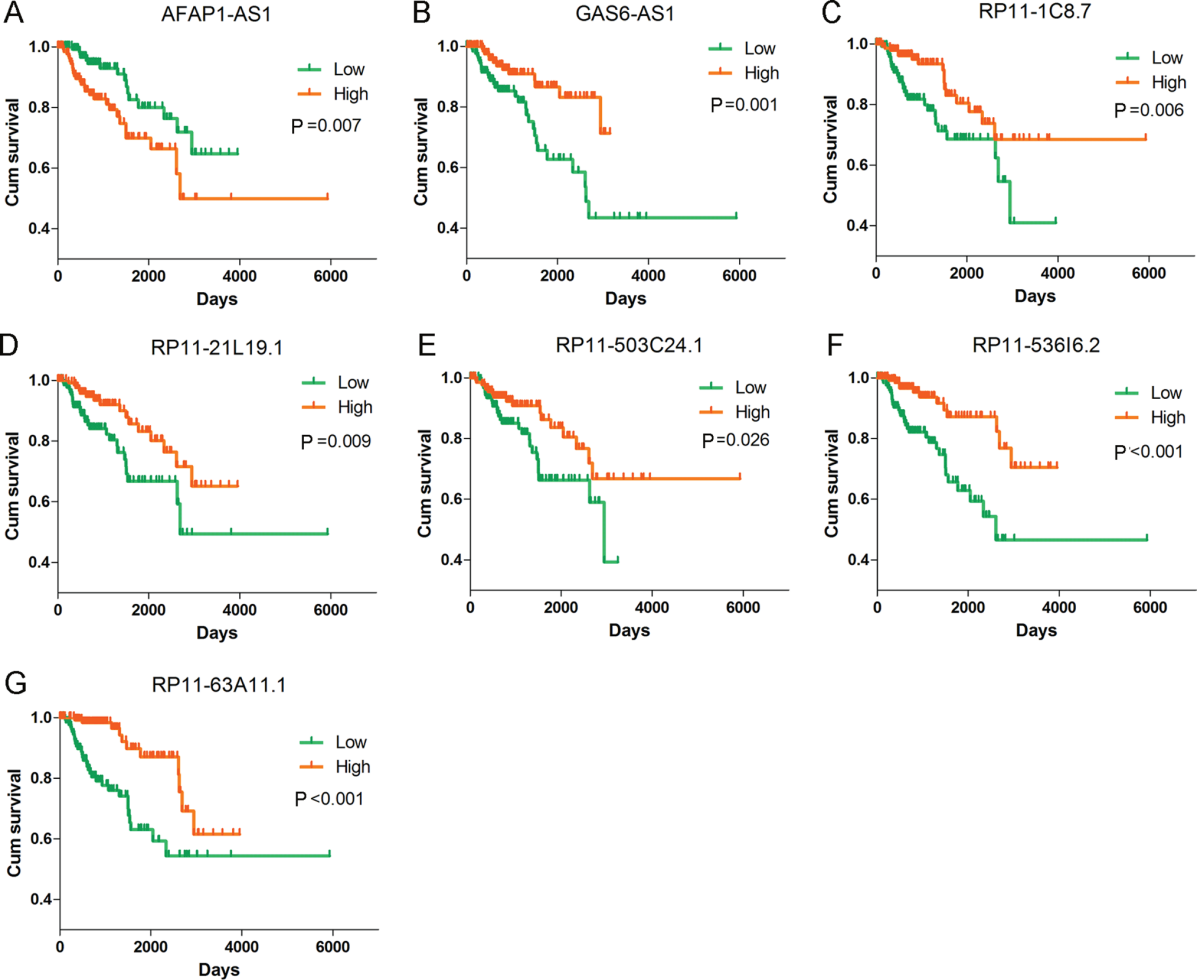
Kaplan–Meier curves for the seven lncRNAs signature in kidney renal papillary cell carcinoma (KIRP) Kaplan-Meier survival curves of the seven lncRNAs showed overall survival outcomes by relative high-risk and low-risk patients. (**A**) AFAP1-AS1 (HR = 1.189, *P* < 0.001); (**B**) GAS6-AS1 (HR = 0.679, *P* < 0.001); (**C**) RP11-1C8.7 (HR = 0.824, *P* < 0.001); (**D**) RP11-21L19.1 (HR = 0.789, *P* < 0.001); (**E**) RP11-503C24.1 (HR = 0.848, *P* = 0.001); (**F**) RP11-536I6.2 (HR = 0.793, *P* < 0.001); (**G**) RP11-63A11.1 (HR = 0.747, *P* ≤ 0.001). Kaplan–Meier curves were generated by GraphPad Prism.

We also evaluated the correlations between PI and each of the clinical features. It was found that PI exhibited moderate ability to foretell the status of tumor stage, metastasis, and lymphatic invasion (Table 4 and Figure 9). The expression of the 7 differentially-expressed lncRNAs in the cohorts of high risk and low risk was illustrated in Figure 10.

**Table 4:** The association of the risk score of the seven lncRNAs signature with clinical features in KIRP patients

Parameters		*N*	*t*-test		ROC		Spearman	
			Mean ± SD	*P*	(AUC)	*P*	r	*P*
**Age**	60≤	143	–5.906 ± 2.007	0.025	0.570	0.040	–0.122	0.040
	>60	142	–6.415 ± 1.714					
**Tumor stage**	T1–2	224	–6.464 ± 1.684	<0.001	0.698	<0.001	0.282	<0.001
	T3–T4	61	–5.015 ± 2.153					
**Lympy node metastasis**	N0	49	–6.207 ± 2.030	<0.001	0.751	<0.001	0.418	<0.001
	N1	28	–4.110 ± 2.426					
**Metasstasis**	M0	95	–6.113 ± 1.886	0.001	0.811	0.002	0.302	0.002
	M1	9	–3.764 ± 1.999					
**Cancer status**	Tumor free	226	–6.493 ± 1.580	<0.001	0.749	<0.001	0.323	<0.001
	With tumor	46	–4.399 ± 2.394					
**TNM stage**	I–II	192	–6.522 ± 1.621	<0.001	0.691	<0.001	0.289	<0.001
	III–IV	66	–5.110 ± 2.159					

### Functional assessment of the differentially-expressed genes in high risk and low risk groups

**Figure 11:**
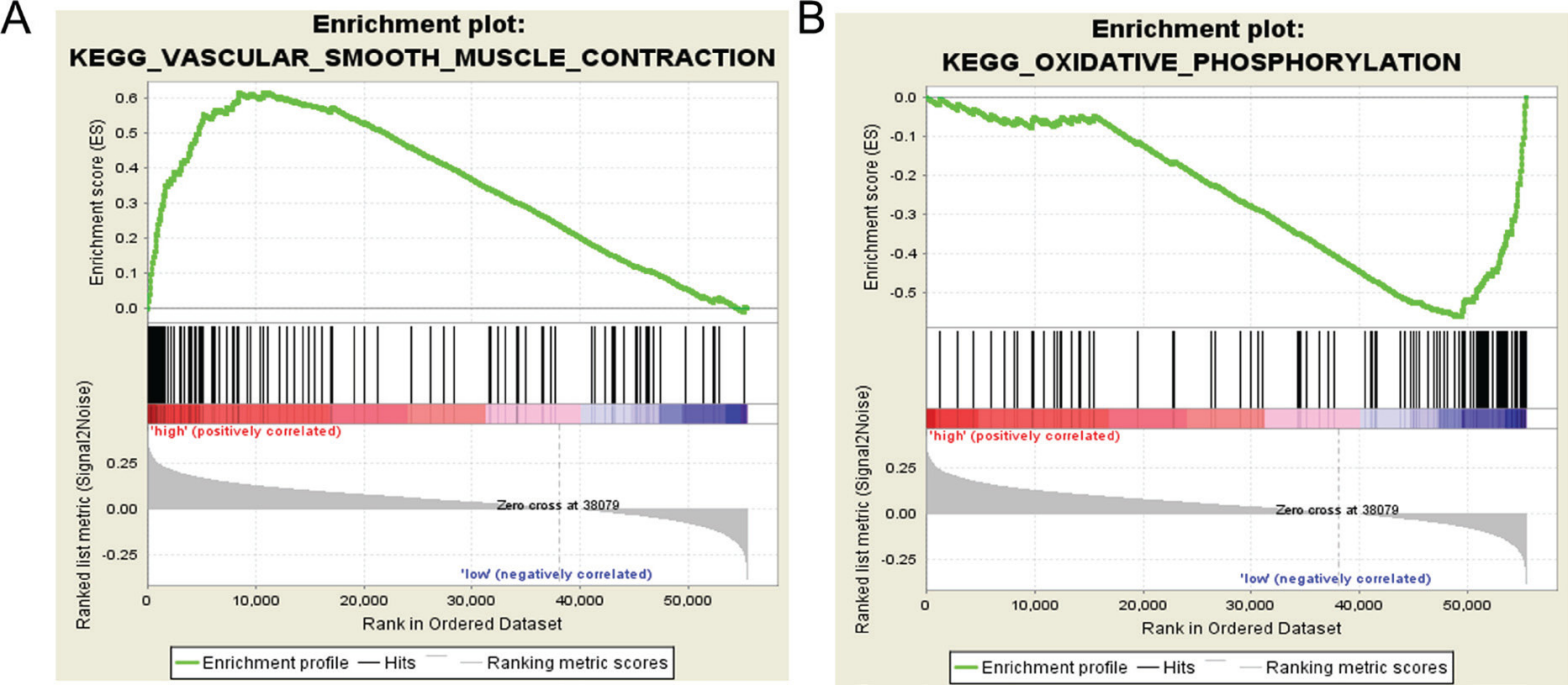
Gene set enrichment analysis revealed KEGG pathways associated with risk score (**A**) KEGG_VASCULAR_SMOOTH_MUSCLE_CONTRACTION; (**B**) KEGG_OXIDATIVE_PHOSPHORYLATION.

**Figure 12:**
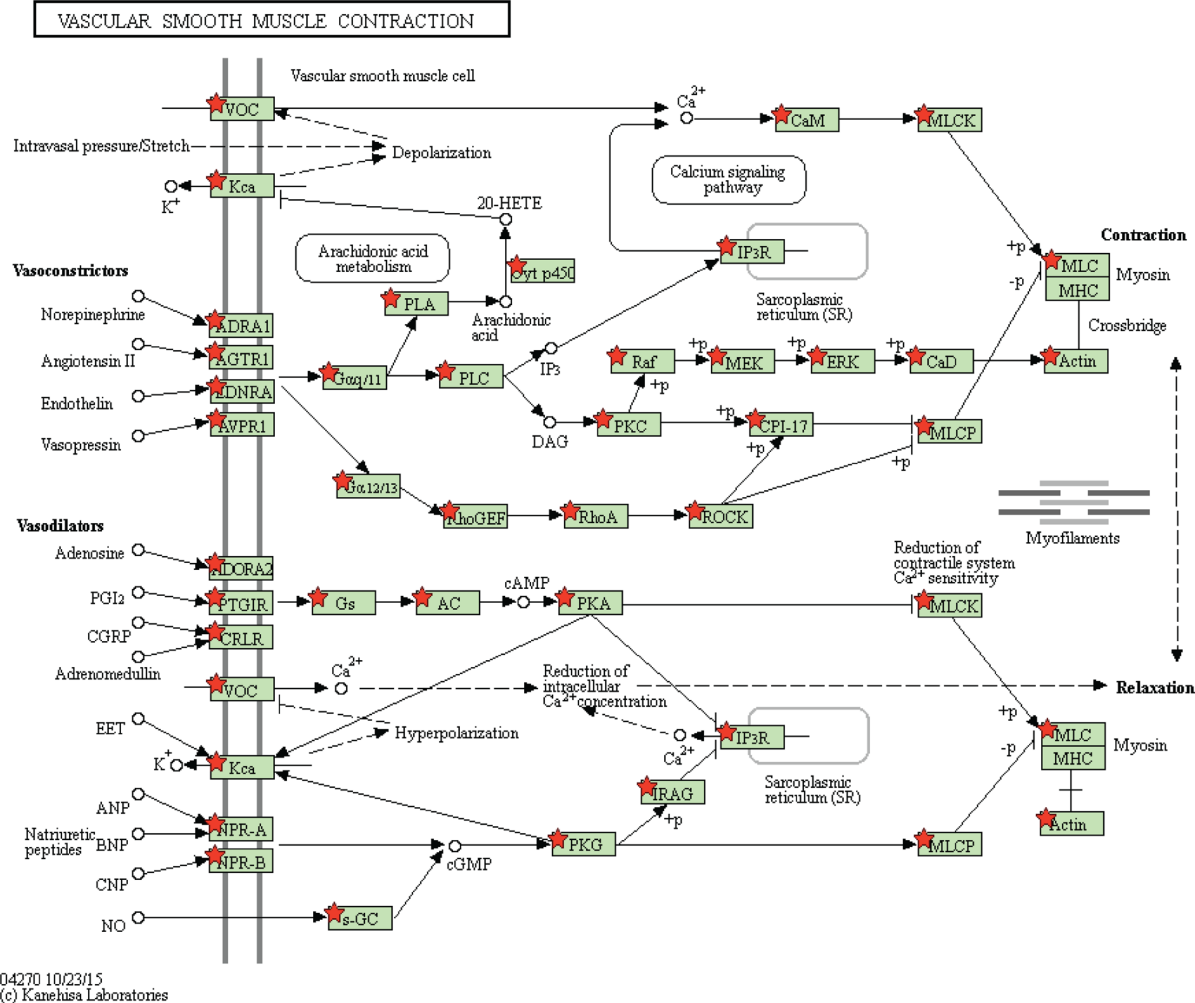
Adapted from KEGG oxidative phosphorylation pathway GSEA enriched genes are labeled in the KEGG pathway with red five pointed stars.

**Figure 13:**
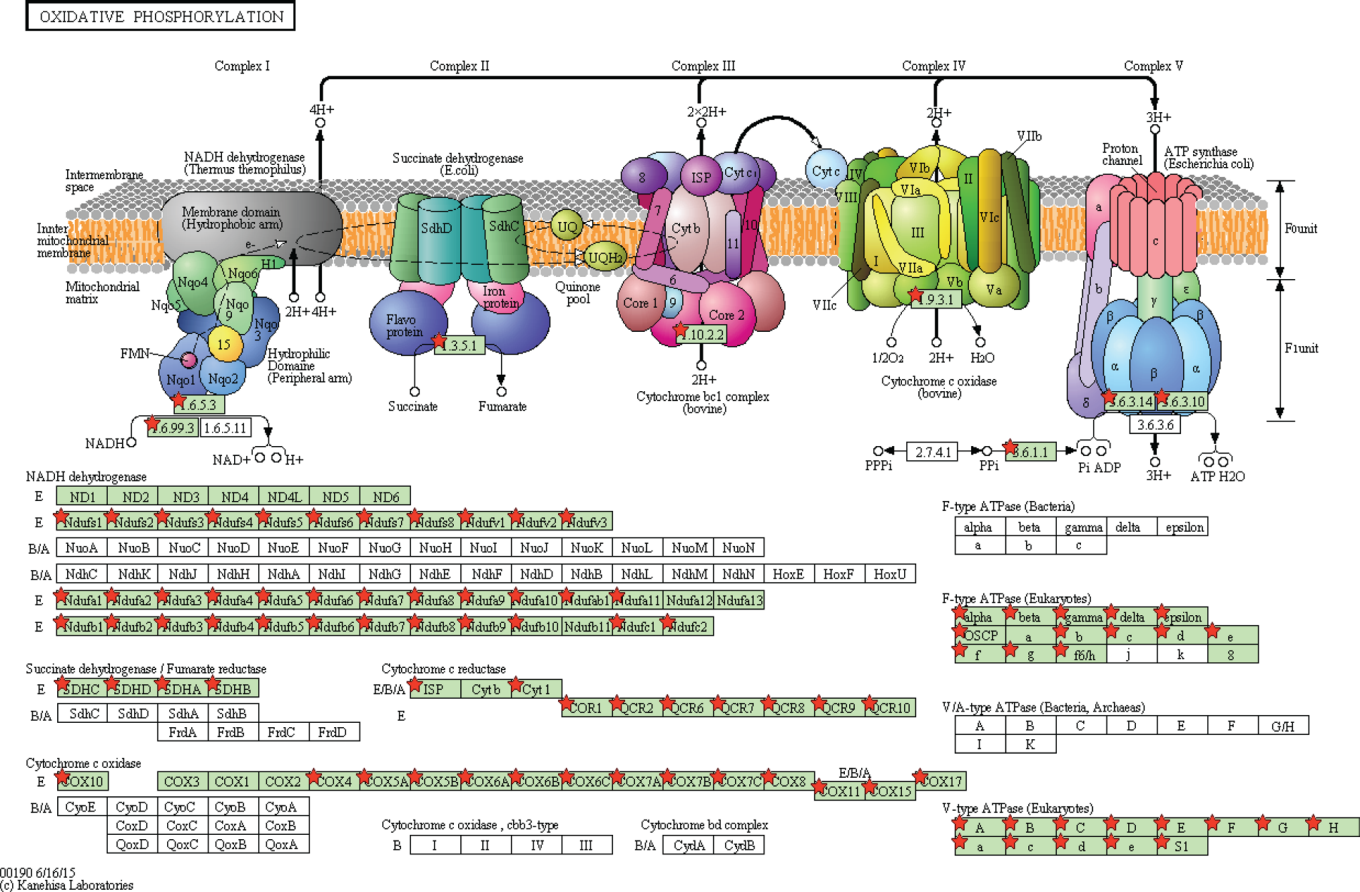
Adapted from KEGG vascular smooth muscle contraction pathway GSEA enriched genes are labeled in the KEGG pathway with red five pointed stars.

Gene Set Enrichment Analysis (GSEA) was performed to distinguish relevant biological processes and signaling pathways [37]. We also investigated differences in the gene expression pattern between KIRP patients and KIRC patients. The gene set enrichment analysis was carried out on the gene sets that showed notably differential expression based on normalized enrichment score (NES) from high to low. It was detected that a total of 156 pathways were considerably enriched in the high risk group, including KEGG_VASCULAR_SMOOTH_MUSCLE_CONTRACTION (Figure 11A, Figure 12), KEGG_TGF_BETA_SIGNALING_PATHWAY, KEGG_MAPK_SIGNALING_PATHWAY. On the contrary, in the low risk group, the enrichment was seen in 21 pathways, including some cancer-related pathways like KEGG_OXIDATIVE_PHOSPHORYLATION (Figure 11B, Figure 13), KEGG_REGULATION_OF_AUTOPHAGY. The relevant top 10 biological pathways were listed in Tables 5 and 6.

**Table 5:** Pathways enriched in high-risk group by using gene set enrichment analysis (GSEA)

NAME	SIZE	ES	NES	NOM	FDR	FWER	RANK AT MAX	LEADING EDGE
				*p*		*p*		
KEGG_VASCULAR_SMOOTH_MUSCLE_CONTRACTION	115	0.62	1.964702	0.002008	0.065481	0.026	11121	tags = 55%, list = 20%, signal = 68%
KEGG_HYPERTROPHIC_CARDIOMYOPATHY_HCM	83	0.59	1.955429	0	0.03834	0.031	9975	tags = 54%, list = 18%, signal = 66%
KEGG_ECM_RECEPTOR_INTERACTION	84	0.67	1.949449	0.002008	0.028178	0.034	9125	tags = 60%, list = 16%, signal = 71%
KEGG_ARRHYTHMOGENIC_RIGHT_VENTRICULAR_CARDIOMYOPATHY_ARVC	74	0.6	1.912274	0.003876	0.034986	0.051	8727	tags = 53%, list = 16%, signal = 62%
KEGG_DILATED_CARDIOMYOPATHY	90	0.58	1.898705	0.001938	0.032262	0.058	8727	tags = 49%, list = 16%, signal = 58%
KEGG_TGF_BETA_SIGNALING_PATHWAY	85	0.62	1.866714	0.001992	0.045007	0.09	11089	tags = 59%, list = 20%, signal = 73%
KEGG_FOCAL_ADHESION	197	0.61	1.855329	0	0.04626	0.098	12655	tags = 60%, list = 23%, signal = 78%
KEGG_OOCYTE_MEIOSIS	110	0.62	1.850145	0.004107	0.044282	0.109	10925	tags = 56%, list = 20%, signal = 70%
KEGG_MAPK_SIGNALING_PATHWAY	265	0.54	1.843136	0.003937	0.044181	0.116	13695	tags = 52%, list = 25%, signal = 69%
KEGG_GAP_JUNCTION	90	0.57	1.835098	0.003861	0.043918	0.121	14440	tags = 58%, list = 26%, signal = 78%

**Table 6:** Pathways enriched in low-risk group by using gene set enrichment analysis (GSEA)

NAME	SIZE	ES	NES	NOM	FDR	FWER	RANK AT MAX	LEADING EDGE
				*p*		*p*		
KEGG_OXIDATIVE_PHOSPHORYLATION	118	–0.6	–1.42998	0.174603	1	0.663	6202	tags = 50%, list = 11%, signal = 56%
KEGG_PARKINSONS_DISEASE	114	–0.5	–1.36873	0.222441	1	0.726	7601	tags = 49%, list = 14%, signal = 57%
KEGG_HISTIDINE_METABOLISM	28	–0.5	–1.2721	0.232283	1	0.828	5883	tags = 43%, list = 11%, signal = 48%
KEGG_GLYCINE_SERINE_AND_THREONINE_METABOLISM	31	–0.5	–1.24302	0.269461	0.917195	0.85	3778	tags = 35%, list = 7%, signal = 38%
KEGG_BUTANOATE_METABOLISM	34	–0.5	–1.19111	0.314961	0.866483	0.877	5384	tags = 41%, list = 10%, signal = 46%
KEGG_VALINE_LEUCINE_AND_ISOLEUCINE_DEGRADATION	44	–0.5	–1.06689	0.470472	1	0.924	5444	tags = 43%, list = 10%, signal = 48%
KEGG_HUNTINGTONS_DISEASE	174	–0.4	–1.06527	0.422179	0.889884	0.925	6292	tags = 35%, list = 11%, signal = 39%
KEGG_PRIMARY_BILE_ACID_BIOSYNTHESIS	16	–0.4	–1.02939	0.41358	0.858544	0.935	5049	tags = 38%, list = 9%, signal = 41%
KEGG_ALZHEIMERS_DISEASE	158	–0.3	–0.95915	0.492126	0.910816	0.957	6202	tags = 35%, list = 11%, signal = 39%
KEGG_REGULATION_OF_AUTOPHAGY	34	-0.3	-0.95755	0.519588	0.822838	0.957	5966	tags = 38%, list = 11%, signal = 43%

### Different signaling pathways in high-risk and low-risk groups

In the high-risk group, 3502 differentially expressed genes (DEGs) were obtained, including 2269 up-regulated and 1233 down-regulated genes (Supplementary Figure 1A, Supplementary Figure 2A). Meanwhile, in the low-risk group, 3808 DEGs were achieved, including 1607 up-regulated and 2201 down-regulated (Supplementary Figure 1B, Supplementary Figure 2B). Omitting those common DEGs between high-risk and low-risk groups, a total of 1081 DEGs were gathered from high-risk group, while 1387 DEGs were collected from low-risk group (Supplementary Figure 1C), which could help explain the different clinical outcomes of these two groups. The results of the KEGG analysis and of the top 50 pathways from GO analysis were presented in Supplementary Figure 3, Supplementary Figure 4 and Supplementary Figure 5.

### Validation of the novel lncRNAs using Gene Expression Omnibus (GEO) DataSets

**Figure 14:**
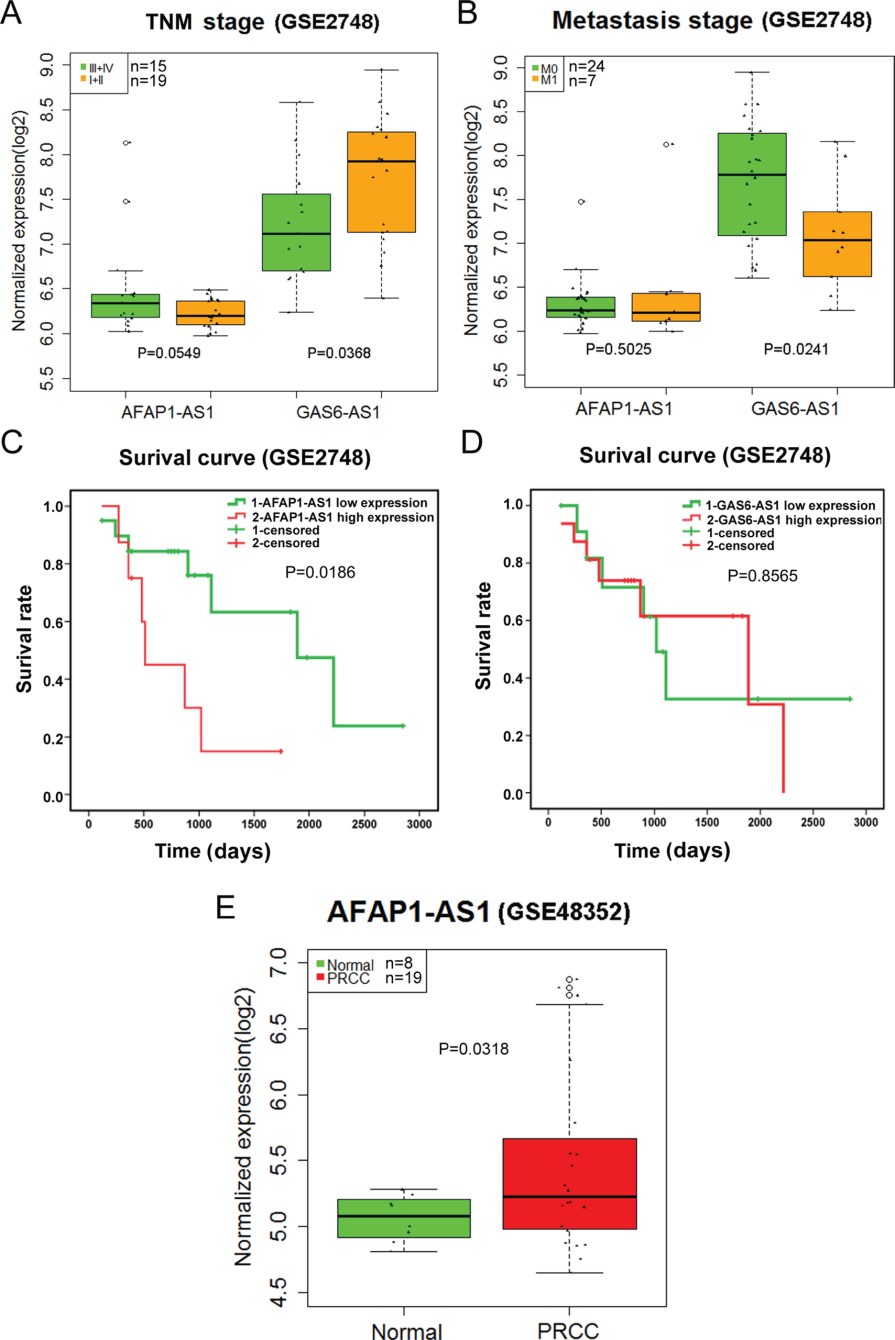
Validation of AFAP1-AS1 and GAS6-AS1 in papillary renal cell carcinoma (PRCC) based on Gene Expression Omnibus (GEO) (**A**) The relationship between the expression of two lncRNAs and TNM stage (III/IV vs. I/II) in papillary renal cell carcinoma (PRCC); (**B**) The relationship between the expression of two lncRNAs and metastasis (M0 vs. M1) in PRCC; (**C**) Kaplan-Meier survival curves of AFAP1-AS1 expression; (**D**) Kaplan-Meier survival curves of GAS61-AS1 expression; (**E**) boxplot of AFAP1-AS1 (GSE48352).

A total of 67 series were retrieved from GEO DataSets according to the search term. Finally, only GSE2748 and GSE48352 were found to contain AFAP1-AS1 or GAS6-AS1 expression. The expression level of AFAP1-AS1 from GSE48352 was higher in papillary renal cell carcinoma (PRCC) than in that of normal controls (*P* = 0.0318) (Figure 14E). The GAS6-AS1 statistical significance differences were noted in the TNM stage (III/IV vs. I/II) and distant metastasis (M0 vs. M1) (all *P* < 0.05) (Figure 14A, 14B). AFAP1-AS1 could be used as a prognostic factor, and GAS6-AS1 might be a protective factor in PRCC (Figure 14C, 14D). These results conformed to our previous findings based on TCGA.

## DISCUSSION

In the current study, we analyzed lncRNA-sequencing data of KIRP in TCGA, and used multivariate Cox regression analysis to obtain seven lncRNAs to form a pool, which included AFAP1-AS1, GAS6-AS1, RP11-1C8.7, RP11-21L19.1, RP11-503C24.1, RP11-536I6.2, and RP11-63A11.1. The PI was also calculated. Surprisingly, the PI was discovered to be an independent indicator for KIRP with high prognostic significance. Additionally, after categorizing the KIRP patients into high risk and low risk groups according to the PI, we observed alterations in key genes and pathways between these two cohorts. To our knowledge, this current study pioneered research into the prognostic value of lncRNAs in KIRP.

RCC represents over 90% of renal carcinomas, among which the subtype KIRP ranked second in the incidence rate, following KIRC [1–5]. Remarkable developments have been achieved in the therapy for metastatic KIRC over the past decades, and a number of targeted therapies have been proposed for it. On the other hand, studies aimed at controlling the metastatic non-clear cell RCC (nccRCC) still remains limited. In addition to KIRP, non-clear cell diseases entail chromophobe and sarcomatoid RCC as well [2]. Currently, some genes and microRNA have been demonstrated to play a role in the prognosis of KIRP. Furthermore, there is a growing awareness that lncRNAs may also exert prognostic capability. However, we know little of the clinical significance of lncRNAs in KIRP despite their multiple functions as a type of non-coding RNA.

In recent years, researchers have had a great opportunity to identify a wide range of novel biomarkers for various malignancies thanks to TCGA, but the roles of lncRNAs in kidney cancers were rarely reported. In 2015, Malouf GG *et al.* [31] conducted an analysis of the RNA-sequencing investigation of 475 primary KIRC cases from TCGA. Four lncRNA subtypes in KIRC were investigated by unsupervised clustering, which correlated with different clinical, pathological, and genomic characteristics of KIRC. The most aggressive tumors were determined via Cluster C2 (23.4%), with the highest Fuhrman grade, the most advanced TNMstage, and the worst overall survival duration. Moreover, enrichment of cluster C2 was carried out for 9p deletion and chromatin remodeler BAP1 somatic mutations. It was interesting to note that cluster C4 (7.8%) was linked with the tumor subtype that was derived from the distal tubules of the nephron. Due to its distinguishable ontogeny, cluster C4 lacked typical alterations in KIRC, but had frequent 1p deletion and 17q gain, and was enriched for MiTF/TFE translocations. However, this was merely a research dealing with KIRC. Because of the histological distinctions between KIRC and KIRP and their different molecular mechanisms, there was a pressing need for the expression profiling of lncRNAs in KIRP and a selection of lncRNAs that could predict progression and prognostication. However, studies on these issues have not been conducted yet.

We singled out the differentially-expressed lncRNAs and then gathered 7 lncRNAs by univariate and multivariate Cox proportional hazards regression, each of which would bear clinical significance such as TNM stage, distant metastasis, and tumor stage, etc. to some extent. More interestingly, the PI composed of these seven lncRNAs displayed considerable predictive potential for disease progression, and could even qualify itself as an independent indicator for prognosis with its clinical significance. Furthermore, taking GEO dataset into validation, we collected 67 KIRP-related series. The clinical and prognostic value of lncRNAs AFAP1-AS1 and GAS6-AS1 could be partially verified by GSE2748 and GSE48352. However, the clinical efficacy of these lncRNAs and the PI required further confirmation with a larger sample size and joint effort.

Having classified the patients into high risk and low PI cohorts according to their PI scores, we examined the signaling pathways of differentially-expressed genes in the two cohorts and found that the major pathways of the high risk group included KEGG_VASCULAR_SMOOTH_MUSCLE_CONTRACTION, KEGG_TGF_BETA_SIGNALING_PATHWAY, and KEGG_MAPK_SIGNALING_PATHWAY; these were utterly different from its low counterpart, of which the main pathways involved KEGG_OXIDATIVE_PHOSPHORYLATION and KEGG_REGULATION_OF_AUTOPHAGY. It was assumed that the pathway differences helped clarify the underlying molecular mechanisms which produced contrasting outcomes. Nonetheless, the relationships between these pathways and KIRP required experimental verification.

For the present, the clinical implications and mechanisms of lncRNAs in other diseases appeal to our research team. By literature research, of the seven lncRNAs, we only retrieved reports on AFAP1-AS1 and GAS6-AS1 in other diseases, whereas reports were lacking on RP11-1C8.7, RP11-21L19.1, RP11-503C24.1, RP11-536I6.2, and RP11-63A11.1. It was thus necessary to conduct in-depth investigation.

A large number of *in vitro* and *in vivo* experiments explored the function and molecular mechanism of AFAP1-AS1. The knockdown of AFAP1-AS1 significantly prohibited cell migration and invasion in nasopharyngeal carcinoma (NPC) and lung cancer cells [32, 33]. Silencing of AFAP1-AS1 markedly reduced hepatocellular carcinoma (HCC) cell proliferation and invasion and decreased tumor growth in a murine allograft model *in vivo*. AFAP1-AS1 also promoted the cell proliferation and invasion through up-regulating the RhoA/Rac2 signaling [34, 35]. Silencing AFAP1-AS1 would inhibit the pancreatic ductal adenocarcinoma (PDAC) cell proliferation, migration, and invasion, while aberrantly-expressed AFAP1-AS1 accelerated cell proliferation, migration, and invasion [36]. The knockdown of AFAP1-AS1 decelerated the intrahepatic cholangiocarcinoma (CCA) cell proliferation and migration [37, 38]. In addition, silencing AFAP1-AS1 prohibited cell migration in part due to reduced expression of MMP-2 and MMP-9 [38]. The knockdown of AFAP1-AS1 prohibited gastric cancer (GC) SGC7901 cell proliferation and induced apoptosis through PTEN/p-AKT pathway [39]. AFAP1-AS1 accelerated ovarian cancer cell proliferation, and AFAP1-AS1 knockdown significantly promoted ovarian cancer cell and esophageal squamous cell carcinoma (ESCC) cell apoptosis [40, 41]. AFAP1-AS1 depletion suppressed SW480 cell and colorectal carcinoma (CRC) cell proliferation and colony formation [42, 43].

Clinical researche into lncRNA AFAP1-AS1 in multiple tumors was diversified. AFAP1-AS1 might act as therapeutic targets in NPC, and anti-PD-1 immune treatment was considered suitable for patients with co-expression of AFAP1-AS1 and PD-1 [32, 44]. Meanwhile, another study also revealed that circulating AFAP1-AS1 possibly played the role of a serum biomarker for NPC determination and outcome prediction after treatment [45]. AFAP1-AS1 was connected with a poor prognostication of HCC [34]. It was demonstrated by microarray analysis that increased expression of AFAP1-AS1 in PDAC tissues suggested lymph node metastasis, perineural invasion, and undesirable survival. When AFAP1-AS1 was used as a prognosis biomarker, the areas under receiver operating characteristic (ROC) curves were 0.8669 and 0.9370 for the forecast of tumor growth during 6 months and 1 year, respectively [36]. It was detected that AFAP1-AS1 showed an upward trend in intrahepatic CCA, and overexpression of AFAP1-AS1 was related to shorter overall survival duration [37, 38]. The up-regulation of AFAP1-AS1 was found in GC tissues as well as GC cells [39]. Additionally, higher expression of AFAP1-AS1 was also discovered in ovarian cancer, gallbladder cancer (GBC), lung adenocarcinoma (LUAD), esophageal squamous cell carcinoma (ESCC) , colorectal carcinoma (CRC), and the highly-expressed AFAP1-AS1 was clearly linked with unsatisfactory prognosis [33, 40–43, 46–49].

Owing to the extensive research on AFAP1-AS1, Liu *et al.* [50] performed meta-analysis on the clinical role of AFAP1-AS1. Web-based search in PubMed, EMBASE, Cochrane Library, China National Knowledge Infrastructure (CNKI) and Wanfang database was conducted. After gathering papers concerned with AFAP1-AS1, Liu *et al.* [50] investigated the relationships between expression levels of AFAP1-AS1 and lymph node metastasis, distant metastasis, overall survival, relapse-free survival, and progression-free survival duration. Eventually, 1,017 patients from eight studies were involved, up to 2015. It was found that high expression of AFAP1-AS1 rendered cancer patients more vulnerable to lymph node metastasis and distant metastasis. Additionally, compared with low expressions of AFAP1-AS1, a significant relationship between highly-expressed AFAP1-AS1 and shorter overall survival, undesirable progression-free survival, and depressive recurrence-free survival was noted. Overall, increased expression of AFAP1-AS1 indicated adverse clinical outcomes. AFAP1-AS1 has high potential to function as a new biomarker for predicting clinical consequences in human malignancies. However, there were no reports on this issue available except our current study. The connections between AFAP1-AS1 and KIRP remained obscure, thus requiring more profound and comprehensive clinical research.

Studies on GAS6-AS1 were also rather scarce, and only relevant studies on lung cancers were retrieved. It was reported that down-regulation of GAS6-AS1 expression was observed in tumor tissues in 50 cases of non-small cell lung cancer (NSCLC) compared with adjacent normal tissues (*P* < 0.001). In addition, reduced expression of GAS6-AS1 was adversely linked with lymph node metastasis (*P* = 0.032) as well as advanced tumor node metastasis stage (*P* = 0.003). Via univariate and multivariate analysis, GAS6-AS1 was considered to act as an independent predictive factor for overall survival duration (*P* = 0.036). Moreover, GAS6-AS1 level showed a negative relationship with GAS6 mRNA level. Aberrant GAS6-AS1 expression could participate in the development of NSCLC via affecting of its host gene, rendering it possible in becoming a diagnosis target in NSCLC patients [51]. The RNA sequencing data analysis revealed that GAS6-AS1 showed a similar trend in KIRP as in NSCLC, as well as being strongly connected with prognosis. Hence, we supposed that GAS6-AS1 might play a vital part in the initiation and development of KIRP, but its molecular mechanism requires further investigation.

Overall, in this current research, we pioneered the deep RNA-sequencing data mining of KIRP lncRNA in TCGA and found that AFAP1-AS1, GAS6-AS1, RP11-1C8.7, RP11-21L19.1, RP11-503C24.1, RP11-536I6.2, and RP11-63A11.1 were differentially expressed in tumor tissues of KIRP, hence exhibiting their capacity to predict outcomes. More importantly, The PI, which consisted of the seven novel lncRNAs, could become a new independent prognostic indicator for KIRP, providing new targets for clinical therapy. Nevertheless, the data in the study were entirely retrieved from TCGA. A joint effort is necessary to clarify the clinical significance and molecular mechanisms of these lncRNAs.

## MATERIALS AND METHODS

### Patients and data mining from TCGA

TCGA has been a major source of valuable data for 33 types of tumors, including for mRNA expression, protein expression, various mutations, amplifications, etc. In this study, we collected sequencing data of KIRP from the TCGA website (https://portal.gdc.cancer.gov/), which contained 321 KIRP tissues and 32 tumor-free adjacent normal tissues up to May 10, 2017. The clinical parameters, including age, tumor size, status of lymph node metastasis, status of distant metastasis, clinical TNM stage, and overall survival, etc. were collected and analyzed. Due to the public availability of the data from TCGA, additional approval by the ethics committee was not required. The data was used and processed according to TCGA Human Subjects Protection and Data Access Policies.

### Evaluation of the differentially-expressed lncRNAs

The current KIPR TCGA dataset was comprised of gene counts for 60,244 mRNAs. In this study, we chose the lncRNAs with descriptions from GENCODE (http://www.gencodegenes.org/) for further analysis, and a subset of expression profiles of 13,198 lncRNAs was eventually obtained. The differentially expressed lncRNAs were subsequently filtered by edgeR R package, with Padj < 0.05 and |log_2_FC| > 2 of expression level when comparing tumor and adjacent normal kidney tissue. The differentially expressed lncRNAs, which had been log2 transformed, were illustrated in a volcano plot and heatmap.

### Construction of DEL-based prognosis index (PI)

In performing the prognosis analysis, we excluded differentially-expressed lncRNAs whose expression levels were below 1 in more than 10% of all subjects [52]. Patients whose key clinical statistics were available in the survival evaluation were involved. Additionally, the follow-up time or survival time of the patients had to exceed 0 days. Subjects without clinical information were removed. The end-point of the current study was overall survival (OS).

The univariate Cox proportional hazards regression was applied to obtain the differentially-expressed lncRNAs that were strongly associated with OS, with the significance level set at 0.05. The multivariate Cox regression model was also used to calculate the prognosis value of these differentially-expressed lncRNAs. Next, the clinical role of these DELs with prognostic value was assessed. Student’s *T-*Test (SPSS Inc., Chicago, IL, USA) was used to examine the differential expression of these lncRNAs between KIRP and non-cancerous kidney tissues. The relationships between DEL expression and clinical progress were evaluated via Student’s *T*-Test, Spearman correlation, and K-M Curve. The PI, which was used for OS prediction, was created on the basis of the linear combination of the expression level multiplied by the regression coefficient that was derived from the multivariate Cox regression model (β). The calculation formula is as follows: PI = βDEL1 × exprDEL1 + βDEL2 × exprDEL2+ ··· + βDELn × exprDELn. [53–55].

Patients with KIRP were categorized into high risk and low risk cohorts according to the cut-off of the individual infection point of PI. The impact of PI on the OS of KIRP cases was measured by univariate and multivariate Cox proportional hazards regression analysis. Several clinical parameters, such as age, tumor stage, and cancer status, etc. were adjusted. Hazard ratio (HR) and 95% confidence intervals (CI) were evaluated. In order to gauge the accuracy of the prognostic model for time-dependent disease outcomes, we utilized the R package “survival ROC” to conduct ROC curve analysis within 5 years as the defining point [53]. Kaplan-Meier survival curves were employed to calculate OS duration for KIRP patients with predicted high or low PIs.

### Different signaling pathways involved in high risk and low risk cohorts

GSEA, also known as functional enrichment analysis, could facilitate the identification of types of genes or proteins that are over-represented in a large set of genes or proteins which may be connected with disease phenotypes. GSEA distinguishes itself from other pathway analysis owing to its enrichment process–the gene expressions in each cohort are first calculated and then enriched according to their expressions. The calculation is as follows: calculate the enrichment score (ES) that embodies the amount to which the genes in the set are over-represented at either the top or bottom of the list. The Molecular Signatures Database (MsigDB) accommodates a large group of annotated gene sets that can be utilized with most GSEA Software. In the current study, a total of 60,483 genes were inputed for GSEA. Gene sets with a *p*-value less than 0.05 and a false discovery rate (FDR) value <0.25 were deemed significantly enriched. If no significant gene set could be obtained, then the gene sets were listed ascendingly according to the orders of *p*-value and FDR. The results were generated by GSEA.

### Different signaling pathways obtained in high-risk and low-risk groups

DEGs were calculated between high-risk and low-risk groups and normal tissues, respectively. DEGs were identified using the edgeR package with Padj<0.05 and |log_2_FC| > 2. The inconsistent DEGs between high-risk and low-risk groups were sent for further signaling pathways analysis to unveil different possible molecular mechanisms. DAVID database was used for the annotation and visualization of DEGs in different risk groups. GO terms and KEGG pathway of DAVID were considered as significant with Padj < 0.05.

### Validation using gene expression omnibus (GEO) datasets

The correlative microarrays from GEO DataSets were gathered to validate the clinical roles of the seven lncRNAs; the following search terms were adopted: (kidney OR nephridium OR renal) AND papillary AND (cancer OR carcinoma OR tumor OR neoplas^*^ OR malignan^*^ OR adenocarcinoma). The levels of lncRNA expression between different groups were analyzed by Student’s *t*-test. Survival analysis was performed using Kaplan–Meier method.

## SUPPLEMENTARY MATERIALS FIGURES


