# Supplementary material for: Survival prediction of kidney renal papillary cell carcinoma by comprehensive LncRNA characterization
Source: Oncotarget. 2017 Nov 28;8(67):110811–29. doi: 10.18632/oncotarget.22732 (PMC5762286; doi:10.18632/oncotarget.22732)
Supplement: Supplementary file 1 [file oncotarget-08-110811-s001.pdf]

Survival prediction of kidney renal papillary cell carcinoma by comprehensive LncRNA characterization

SUPPLEMENTARY MATERIALS

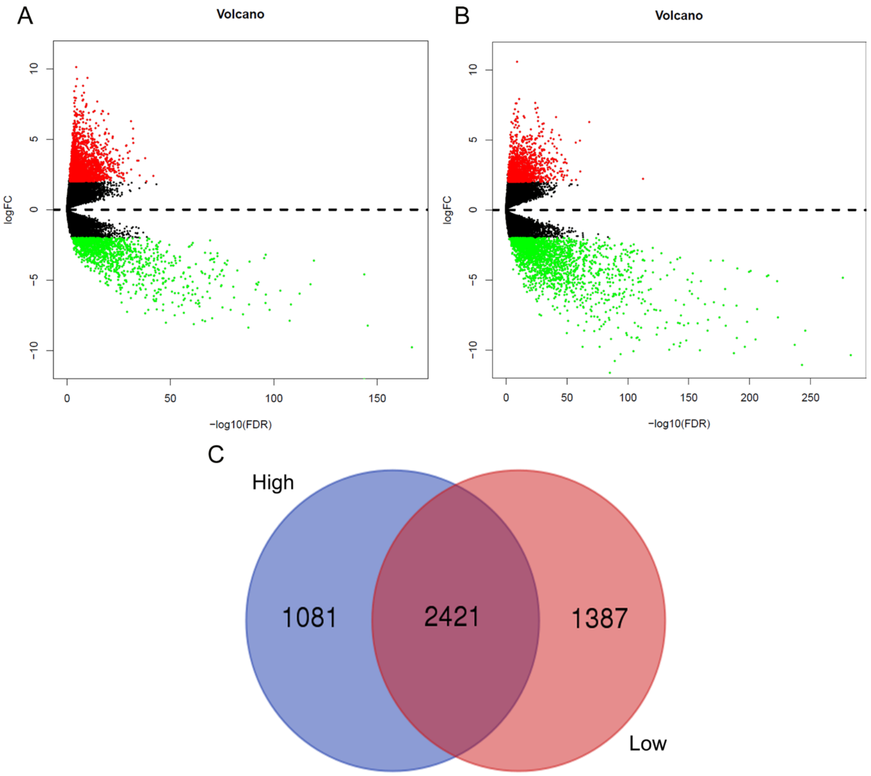

Supplementary Figure 1: Differentially expressed genes (DEGs) analysis. (**A**) Volcano plot of DEGs in high risk score group of kidney renal papillary cell carcinoma (KIRP) and normal kidney samples. (**B**) Volcano plot of DEGs in low risk score group and normal kidney samples. DEGs identified using the edgeR package with Padj < 0.05 and $|\log_2 FC| > 2$. Volcano plot was drawn by the gplots package. (**C**) Venn diagrams of overlapping genes from DEGs. Blue: DEGs from high risk score group of KIRP and normal kidney samples, Light purple: DEGs from low risk score group of KIRP and normal kidney samples, Dark purple: overlapping genes.

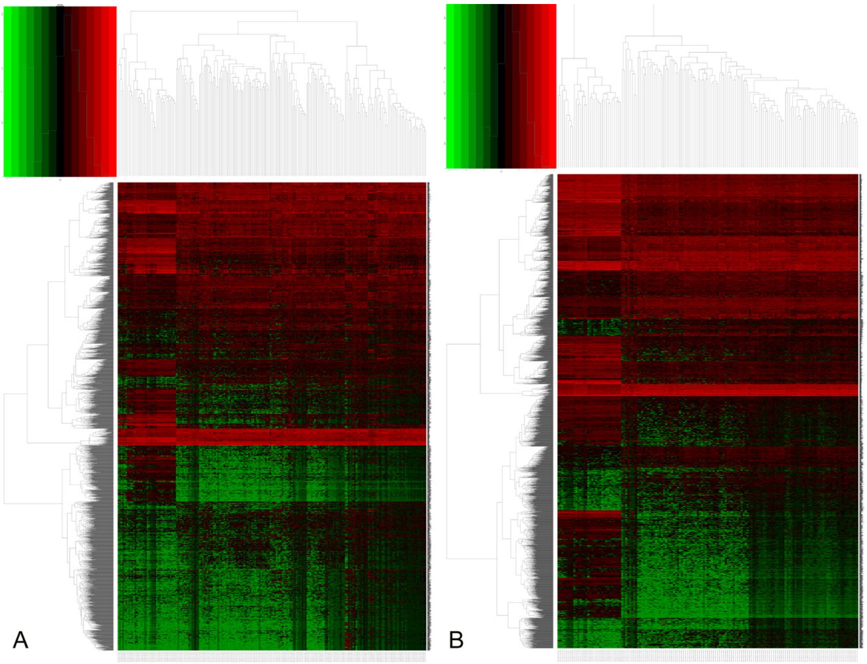

Supplementary Figure 2: Heatmap of differentially expressed genes (DEGs) in kidney renal papillary cell carcinoma (KIRP). (**A**) Heatmap of DEGs in high risk score group of KIRP and normal kidney samples. (**B**) Heatmap of DEGs in low risk score group of KIRP and normal kidney samples. DEGs identified using the edgeR package with Padj < 0.05 and $|\log_2 FC| > 2$. Heatmap was generated by the gplots package.

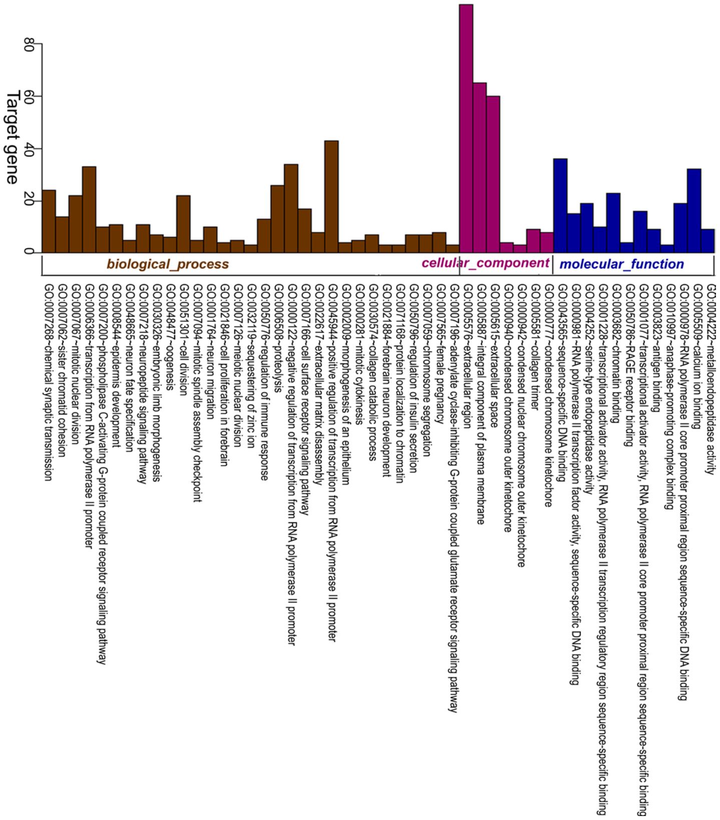

Supplementary Figure 3: Gene Ontology (GO) annotation of 1081 differentially expressed genes in high risk group. The bar plot of GO Annotation was generated by R language with top 50 items. GO Annotation was generated using DAVID online tool.

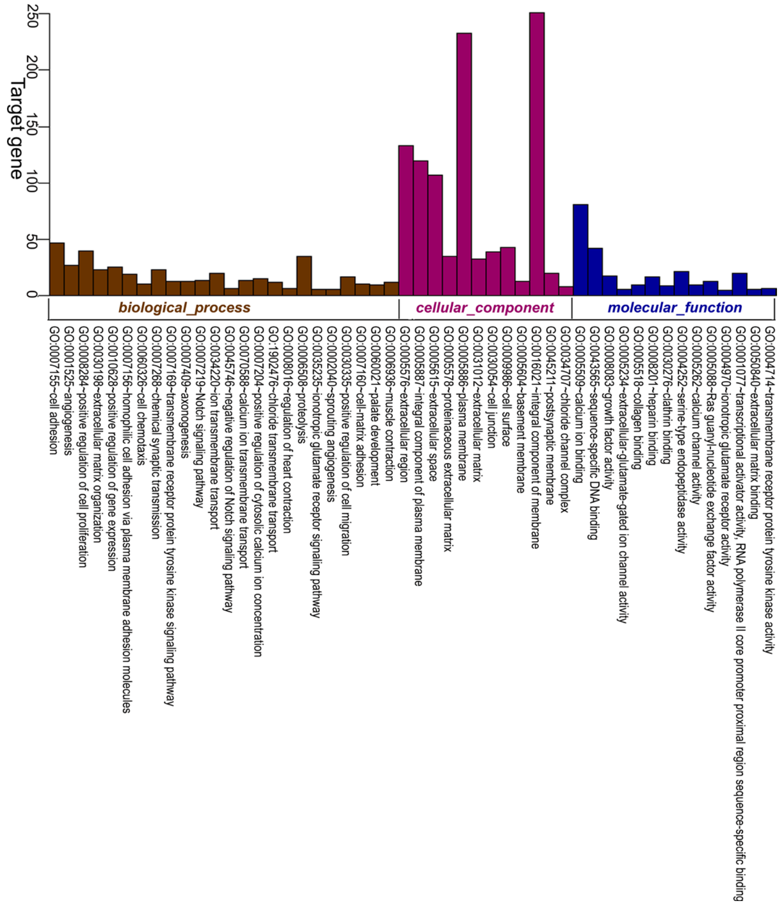

Supplementary Figure 4: Gene Ontology (GO) annotation of 1387 differentially expressed genes in low risk group. The bar plot of GO Annotation was generated by R language with top 50 items. GO Annotation was generated using DAVID online tool.

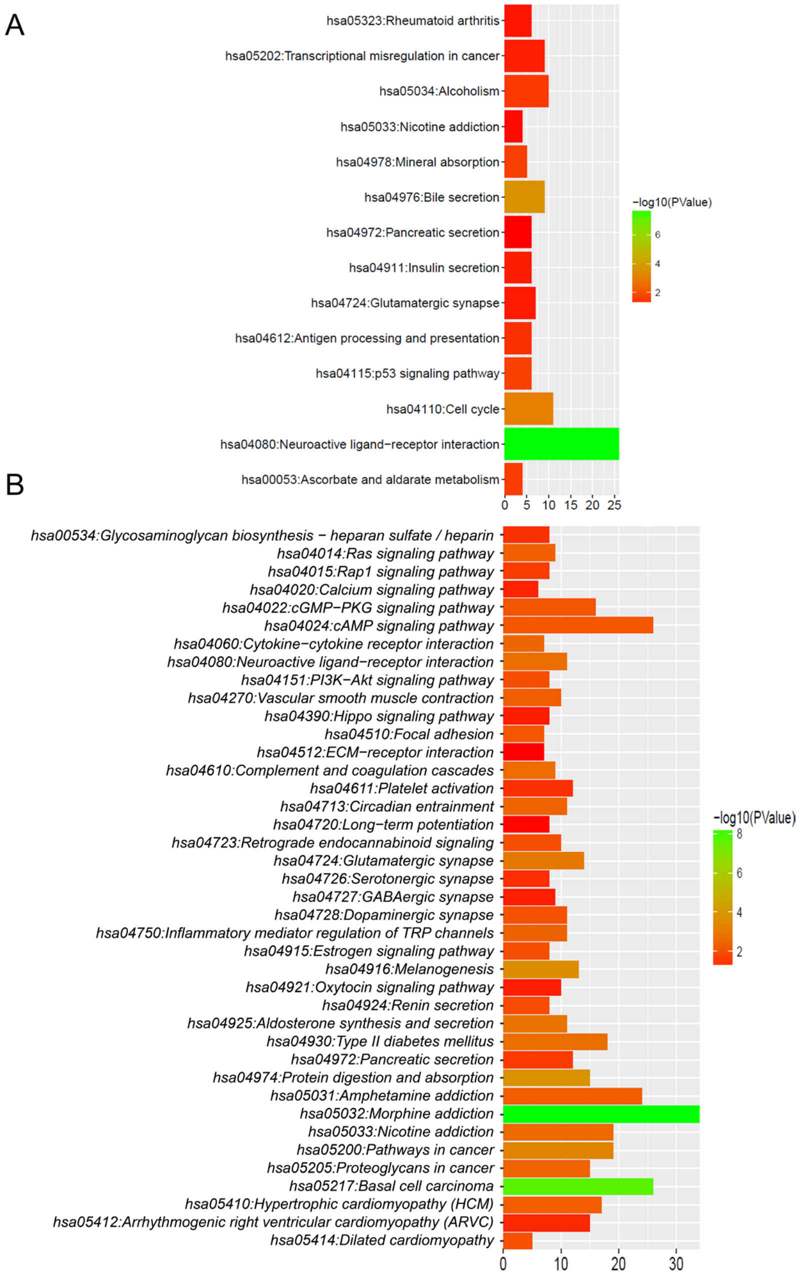

Supplementary Figure 5: Kyoto Encyclopedia of Genes and Genomes (KEGG) pathways. KEGG Pathways were drawn by the ggplot2 package in R language. (**A**) Kyoto Encyclopedia of Genes and Genomes (KEGG) pathways of 1081 differentially expressed genes in high risk group. The horizontal axis showed the number of genes. Column color represented the $-\log_{10}$ (*P*-Value). Green: high degree of enrichment, red: low degree of enrichment. KEGG Pathways were identified by DAVID online tool. (**B**) Kyoto Encyclopedia of Genes and Genomes (KEGG) pathways of 1387 differentially expressed genes in low risk group.
